# Supplementary material for: Transport mechanism of human bilirubin transporter ABCC2 tuned by the inter-module regulatory domain
Source: Nat Commun. 2024 Feb 5;15:1061. doi: 10.1038/s41467-024-45337-5 (PMC10844203; doi:10.1038/s41467-024-45337-5)
Supplement: Supplementary file 1 — Supplementary Information [file 41467_2024_45337_MOESM1_ESM.pdf]

Supplementary

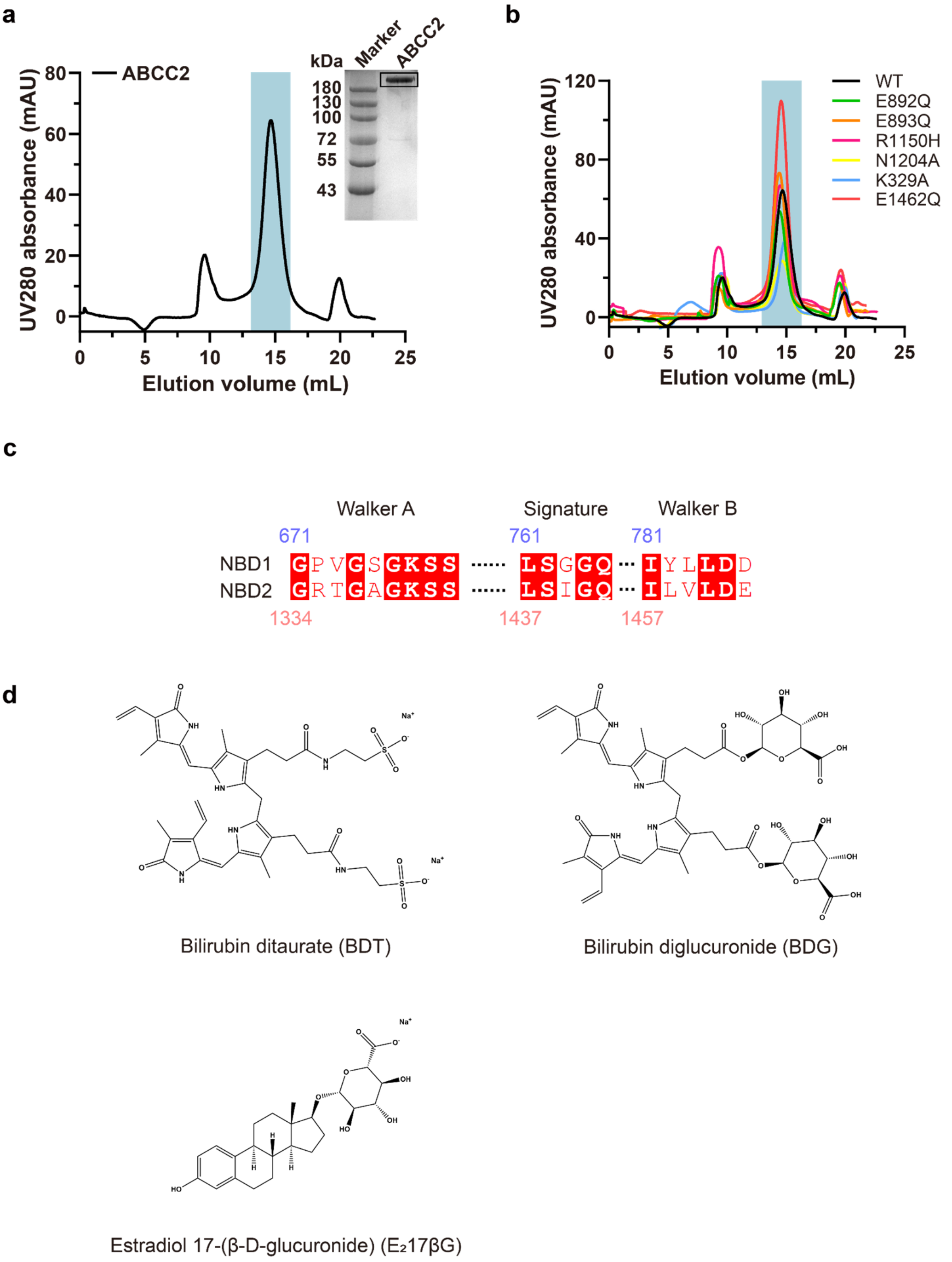

Supplementary Fig. 1 Purification of ABCC2 and mutants.

a Representative size-exclusion chromatography profile and SDS-PAGE analysis of human ABCC2. **b** Size-exclusion chromatography profiles of ABCC2 and mutants. **c** Sequence alignment of the ATP-binding and hydrolysis motifs on NBD1 (consensus site) and NBD2 (degenerate site). **d** Structural formulas of the conjugated bilirubin BDG and its analog BDT, as well as $E_2$17βG.

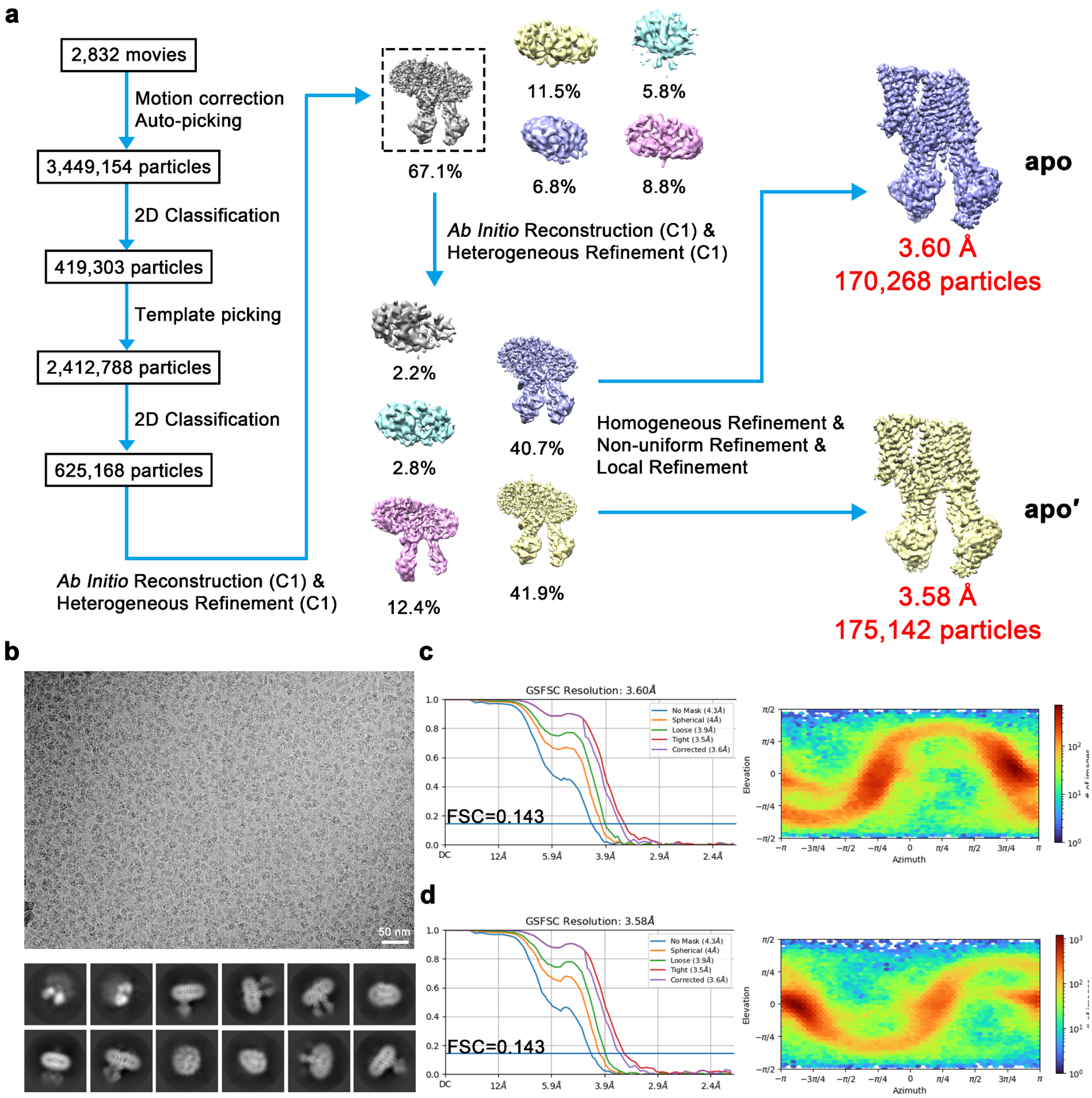

Supplementary Fig. 2 Cryo-EM analysis of apo-form ABCC2.

a Flowchart of the cryo-EM data processing by cryoSPARC 3.1. **b** Representative cryo-EM micrograph and 2D averages. Bar: 50 nm. Fourier shell correlation (FSC) curves and Euler angle distributions of the classified particles used for the final 3D refinement of the overall maps for **c** apo-form and **d** apo′-form.

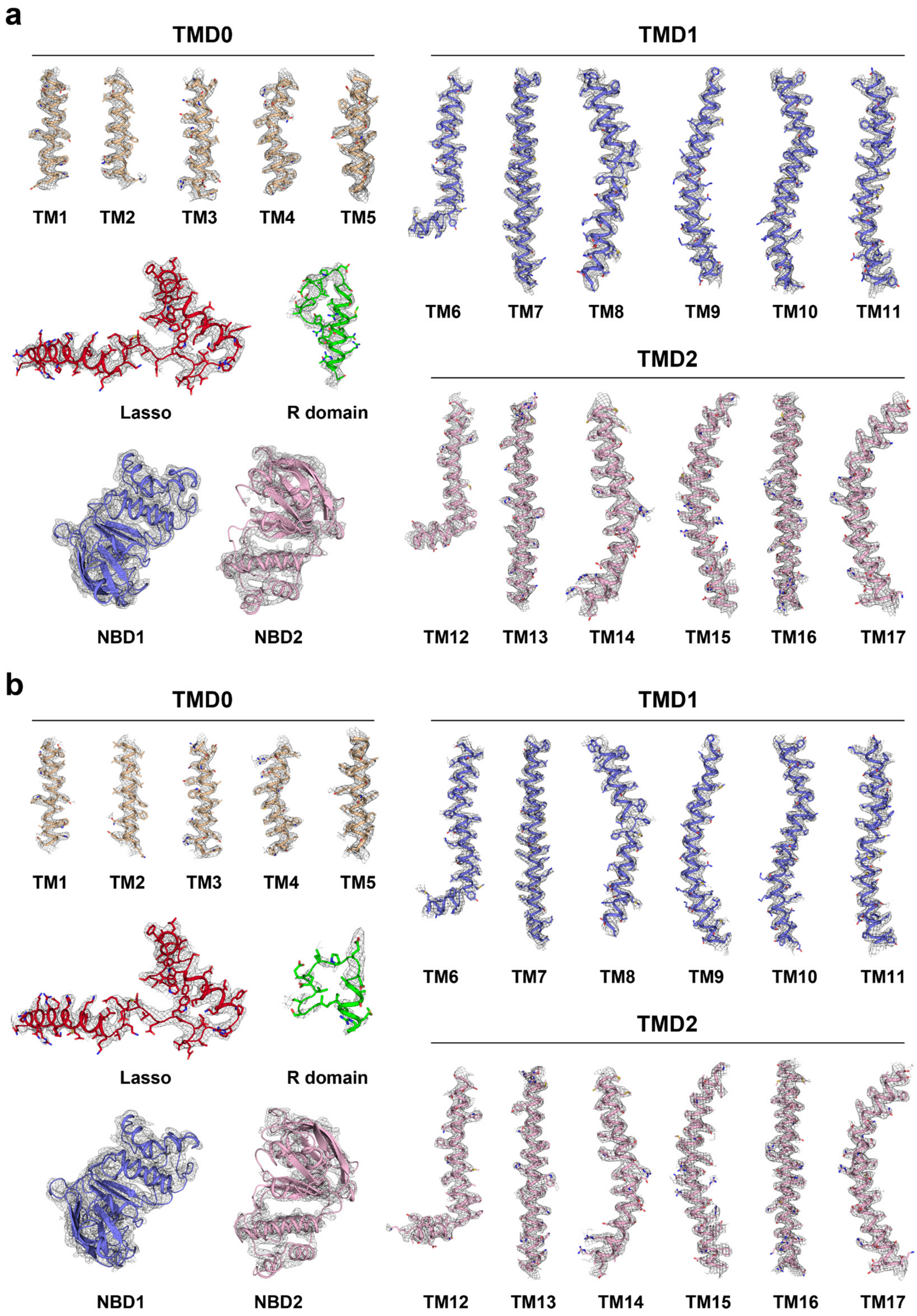

Supplementary Fig. 3 Cryo-EM densities of representative segments of the structure of apo (a) and apo′ (b) ABCC2.

The structures were reconstructed with C1 symmetry. Contour levels are set at 5 σ.

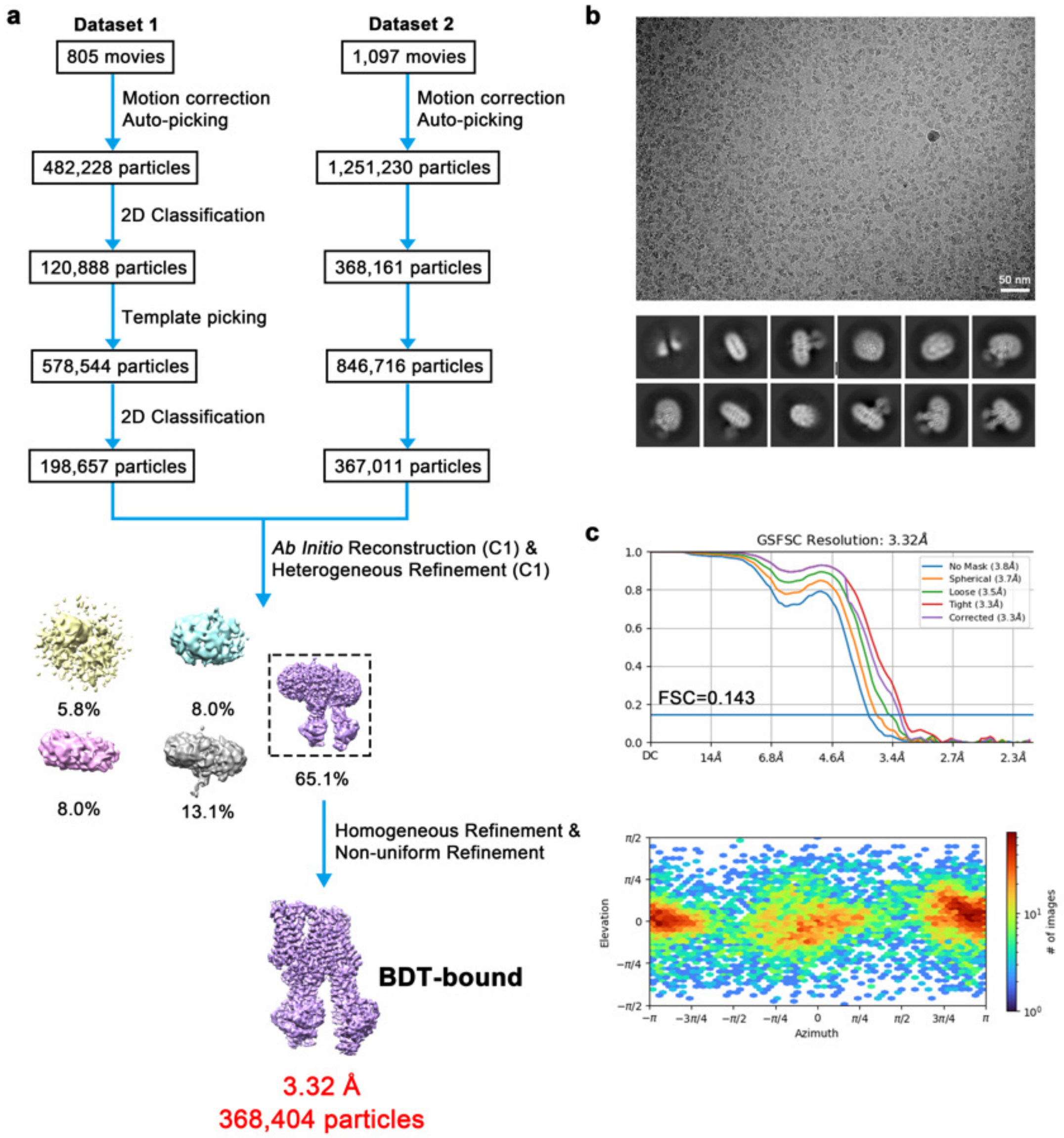

Supplementary Fig. 4 Cryo-EM analysis of BDT-bound ABCC2.

a Flowchart of the cryo-EM data processing by cryoSPARC 3.1. **b** Representative cryo-EM micrograph and 2D averages. Bar: 50 nm. **c** FSC curve and Euler angle distribution of the classified particles used for the final 3D refinement of the overall map for the BDT-bound ABCC2.

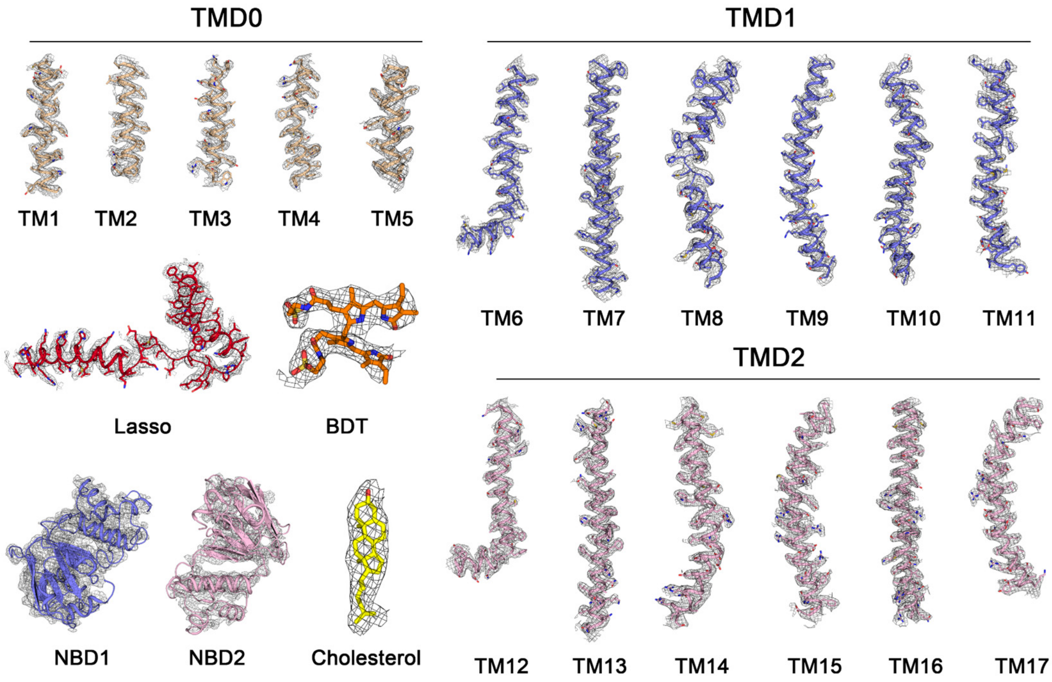

Supplementary Fig. 5 Cryo-EM densities of representative segments of the structure of BDT-bound ABCC2.

The structure was reconstructed with C1 symmetry. Contour level is set at 5 σ.

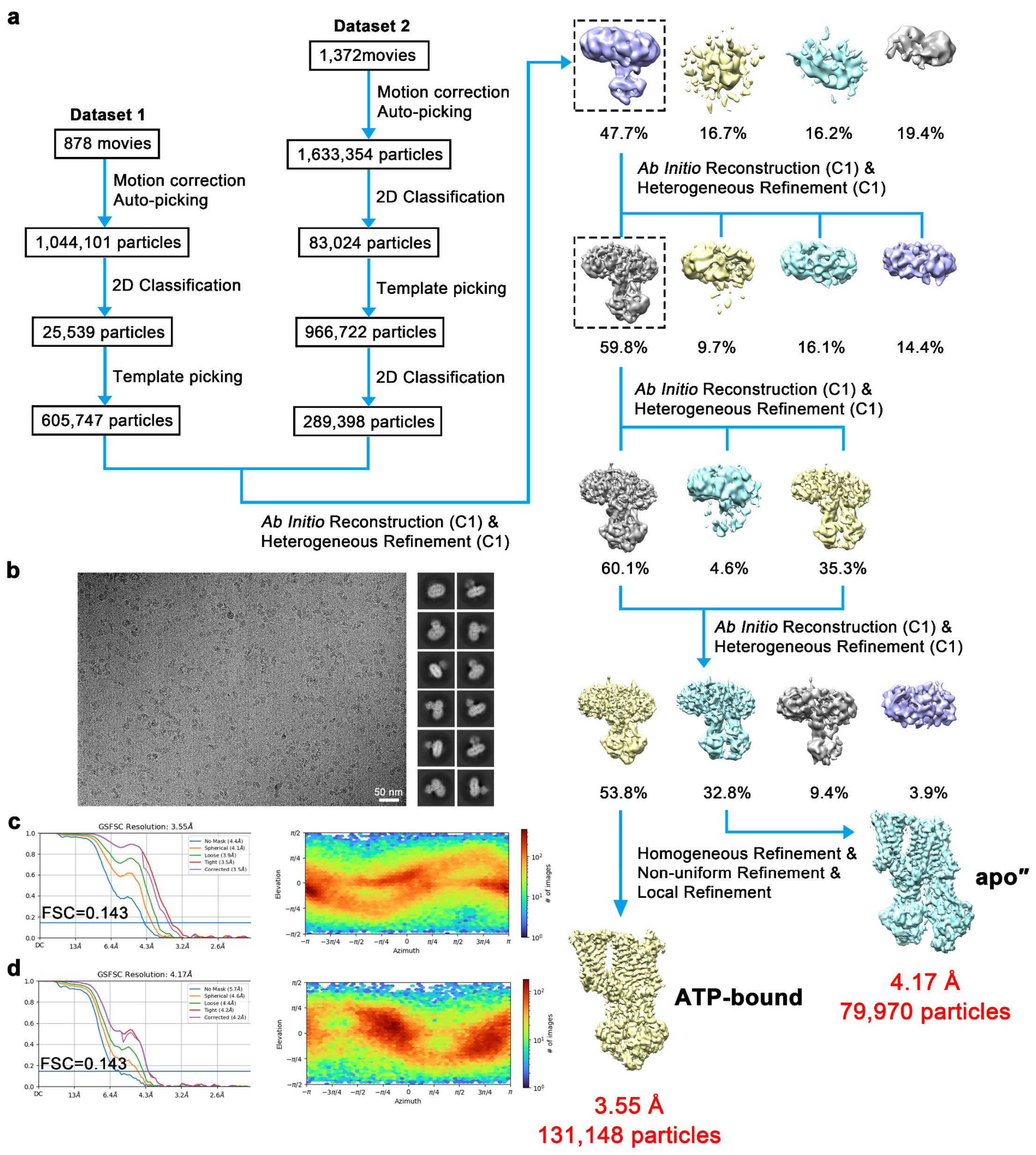

Supplementary Fig. 6 Cryo-EM analysis of ATP/ADP-bound ABCC2.

a Flowchart of the cryo-EM data processing by cryoSPARC 3.1. **b** Representative cryo-EM micrograph and 2D averages. Bar: 50 nm. FSC curves and Euler angle distributions of the classified particles used for the final 3D refinement of the overall maps for **c** ATP/ADP- bound and **d** apo″-form ABCC2.

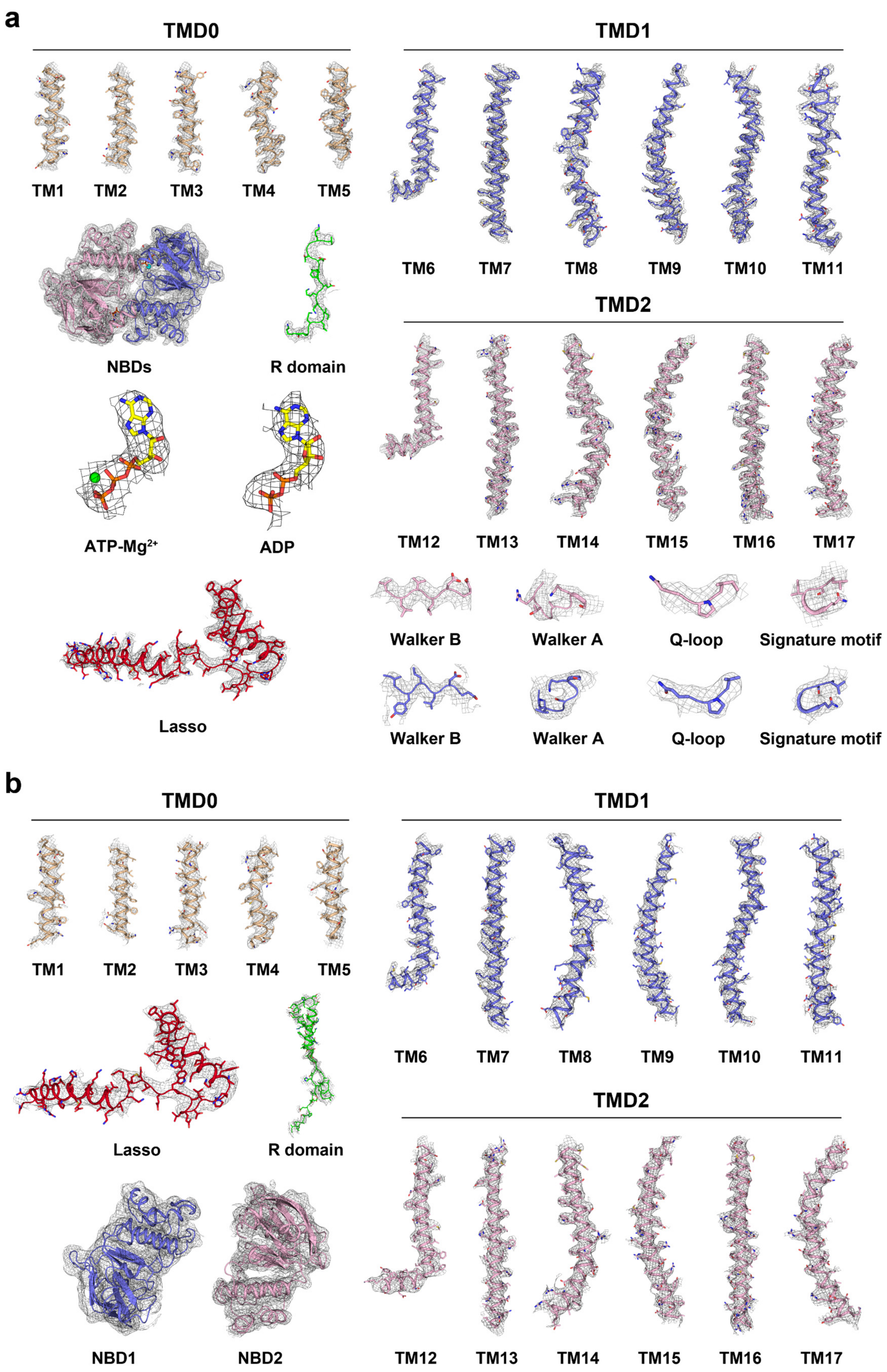

Supplementary Fig. 7 Cryo-EM densities of representative segments of the structures of ATP/ADP-bound ABCC2 (a) and apo″ ABCC2 (b).

The structures were reconstructed with C1 symmetry. Contour levels are set at 5 σ.

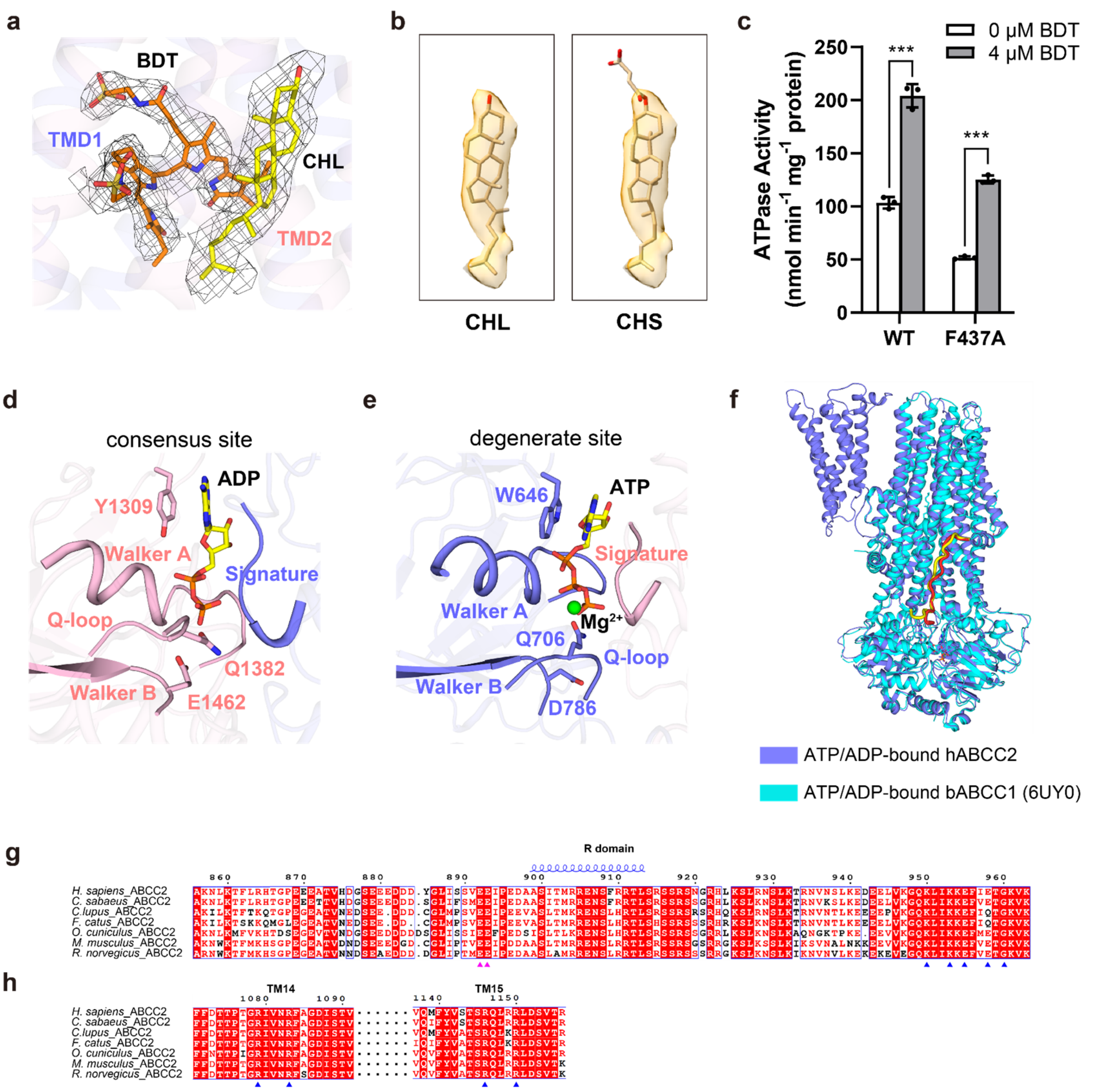

Supplementary Fig. 8 Conformational changes of ABCC2 upon ATP binding.

a Density map for BDT and cholesterol molecules at a contour level of 5 σ colored using the same color scheme as shown in **Fig. 3c**. **b** Comparison of fitting CHL or CHS molecules into the extra density at a contour level of 5 σ, respectively. Carbon atoms are colored in wheat, with oxygen atom colored in red. **c** The ATPase activities of ABCC2 wild type (WT) and F437A mutant in the presence or absence of 4 μM BDT. Each data point is the average of independent experiments (n = 3), and error bars represent the means ± SD. The statistical significance is calculated using unpaired, two-sided *t* tests. *P*-values: WT, 0.000145; F437A < 0.0001. The *P* values of <0.05, 0.01, and 0.001 are indicated with *, **, and ***, respectively. Source data are provided as a Source Data file. **d** and **e** Interaction details of ATPase sites of ATP/ADP-bound ABCC2. **f** Superposition of the ATP/ADP-bound bovine ABCC1 (cyan, PDB: 6UY0) against

ATP/ADP-bound human ABCC2 (slate). R domains from bovine ABCC1 and human ABCC2 are colored in yellow and red, respectively. Multiple-sequence alignments of **g** the R domain and **h** TM14 and TM15, which interact with the C-segment of the R domains of ABCC2 and its homologs. The interacting residues on the M- or C-segments are labeled with pink or blue triangles below the sequence, respectively.

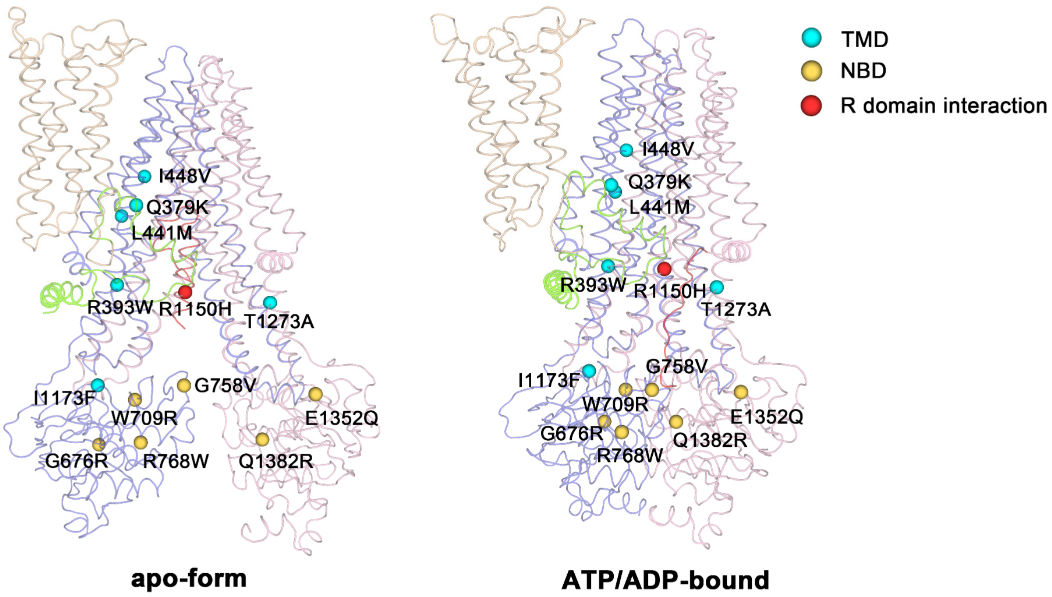

Supplementary Fig. 9 Distribution of Dubin-Johnson syndrome associated pathogenic variants in ABCC2.

The three groups of mutant residues are represented as dots in different colors.

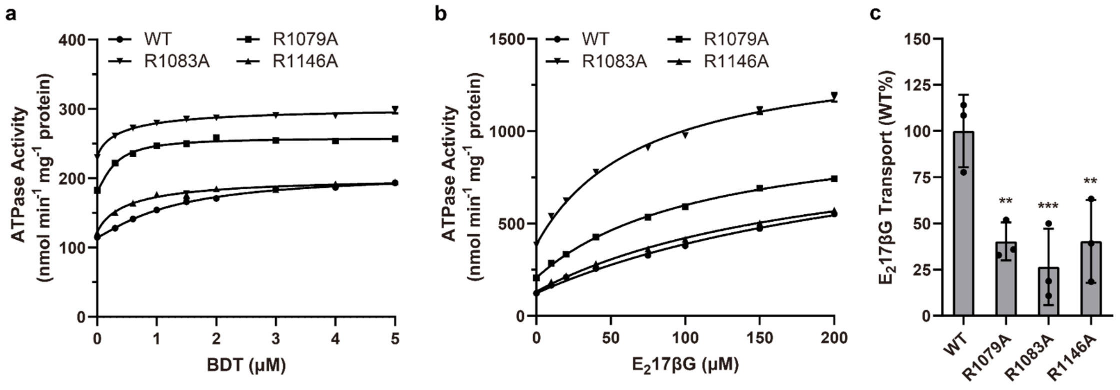

Supplementary Fig. 10 Biochemical assays of R domain anchored on TMD2.

a The BDT- and **b** $E_2$17βG-stimulated ATPase activity assays of ABCC2 and R1079A, R1083A, R1146A mutants. The data points are fitted with the Hill equation in **a** and the Michaelis-Menten equation in **b**. The EC_{50} values towards BDT was determined to be 1.31 ± 0.11, 0.29 ± 0.02, 0.42 ± 0.08 and 0.54 ± 0.10 μM for ABCC2 and R1079A, R1083A and R1146A mutants, respectively. The EC_{50} values towards $E_2$17βG were determined to be 282.8 ± 28.9, 114.5 ± 4.3, 72.0 ± 4.7 and 199.8 ± 12.2 μM for ABCC2 and R1079A, R1083A and R1146A mutants, respectively. All data points for **a** and **b** represent means of three independent measurements (n=3), and error bars represent the means ± SD. **c** The transport activity assays of ABCC2 and R1079A, R1083A, and R1146A mutants using radioisotope-labeled substrate $E_2$17βG. The transport activities of mutants were normalized by WT. Each data point is the average of independent experiments (n = 3), and error bars represent the means ± SD. One-way ANOVA is used for the comparison of statistical significance of WT and mutants. *P*-values: R1079A, 0.0017; R1083A, 0.0002; R1146A, 0.0017. The *P* values of <0.05, 0.01 and 0.001 are indicated with *, ** and ***, respectively. Source data are provided as a Source Data file.

a

Number of fragments detected by LC-MS/MS			
Residue	Phosphorylated	Non-phosphorylated	Efficiency of phosphorylation
T873	92	329	21.8%
S878	190	231	45.1%
S889	14	407	3.3%
S890	14	407	3.3%
S926	6	8	42.9%
S930	7	7	50.0%
T933	1	10	9.1%
S938	10	82	10.9%

b

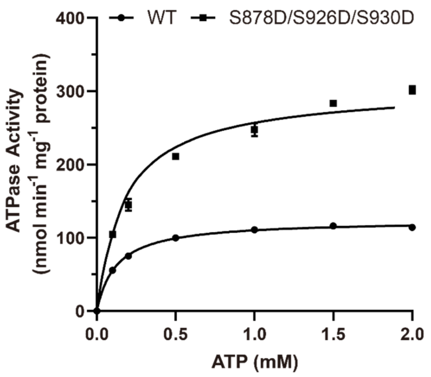

Supplementary Fig. 11 Phosphorylation state of the R domain.

a The phosphorylated residues of the R domain detected by LC-MS/MS. The probabilities of these residues being phosphorylated detected by LC-MS/MS are also listed. **b** The ATPase activities of wild-type ABCC2 and the triple mutation variant (S878D/S926D/S930D) mimicking phosphorylation. The data are fitted using the Michaelis-Menten equation. The purified wild-type (WT) ABCC2 protein and the triple mutation displayed ATPase activities with K_m and V_{max} values of 0.12 ± 0.004, 0.25 ± 0.02 mM and 123.9 ± 0.8, 327.1 ± 0.3 nmol Pi min^{-1} mg^{-1} protein, respectively. Each data point is the average of independent experiments (n = 3), and error bars represent the means ± SD. Source data are provided as a Source Data file.

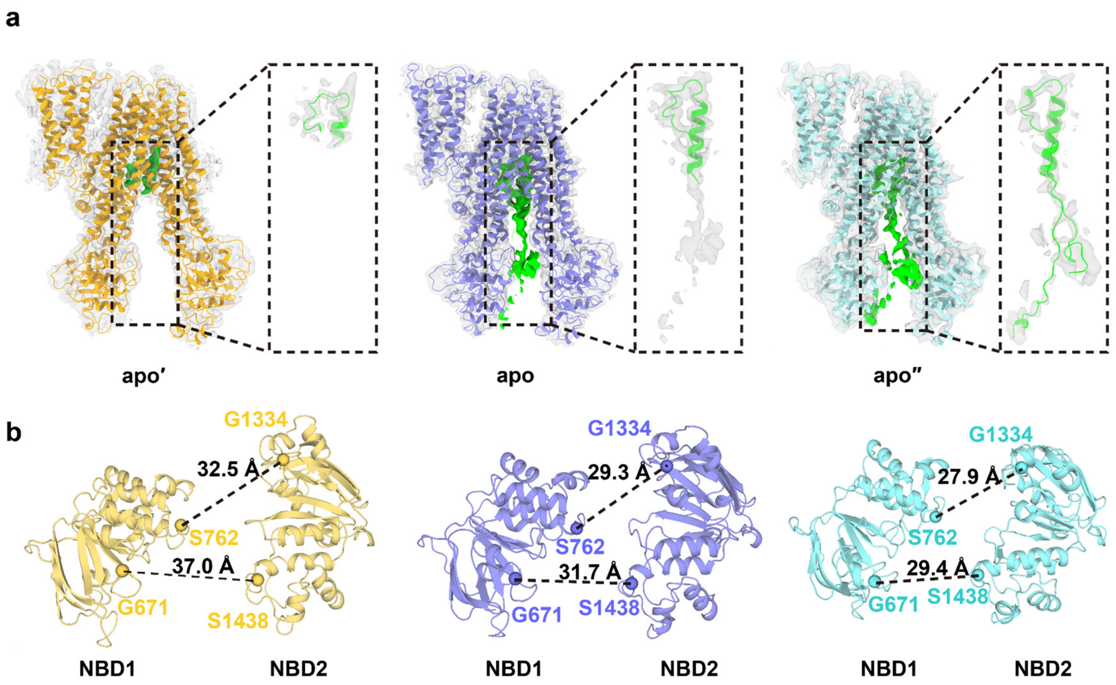

Supplementary Fig. 12 Conformational flexibility of ABCC2 apo forms.

a. Maps and models of the three apo form structures of ABCC2**.** The details of the modeled R domain backbone and corresponding densities are shown by zoom-in images on the right, respectively. The density maps are shown with contour levels at 3.5 σ. **b** The distances between two NBDs in the three apo-form structures.

Supplementary Table 1 Cryo-EM data collection, refinement and validation statistics.

	apo′ ABCC2	apo ABCC2	apo″ ABCC2	BDT-bound ABCC2	ATP/ADP-bound ABCC2
**Data collection and processing**					
Magnification	81,000	81,000	81,000	81,000	81,000
Voltage (kV)	300	300	300	300	300
Electron exposure ($e^-/Å^2$)	55	55	55	55	55
Defocus range (μm)	-2.0 to -1.2	-2.0 to -1.2	-2.0 to -1.2	-2.0 to -1.2	-2.0 to -1.2
Pixel size (Å)	1.07	1.07	1.07	1.07	1.07
Symmetry imposed	C1	C1	C1	C1	C1
Initial particle images (no.)	3,449,154	3,449,154	2,677,455	1,733,458	2,677,455
Final particle images (no.)	175,142	170,268	79,970	368,404	131,148
Map resolution (Å)	3.6	3.6	4.2	3.3	3.6
FSC threshold	0.143	0.143	0.143	0.143	0.143
Map resolution range (Å)	3.1-5.5	3.1-5.5	3.8-6.2	2.8-5.2	3.2-5.2
**Refinement**					
Initial model used (PDB code)					6UY0
Model resolution (Å)	3.6	3.6	4.2	3.3	3.5
FSC threshold	0.143	0.143	0.143	0.143	0.143
Map sharpening B factor ($Å^2$)	-168.5	-167.9	-187.5	-159.2	-155.6
Model composition					
Non-hydrogen atoms	10,994	11,238	11,512	11,111	11,265
Protein residues	1,383	1,416	1,457	1,388	1410
Ligands	None	None	None	A BDT molecule and a cholesterol molecule	An ATP molecule, an ADP molecule and a Mg^{2+}
B factors ($Å^2$)					
Protein	155.89	116.72	163.90	105.06	115.52
Ligand	None	None	None	60.19	123.39
R.m.s. deviations					
Bond lengths (Å)	0.003	0.003	0.003	0.003	0.004
Bond angles (°)	0.551	0.647	0.655	0.556	0.583
Validation					
MolProbity score	1.56	1.55	1.69	1.23	1.75
Clashscore	6.92	8.89	12.01	4.54	7.60
Poor rotamers (%)	0.00	0.00	0.00	0.41	0.16
Ramachandran plot					
Favored (%)	96.95	97.66	97.52	98.48	95.23
Allowed (%)	2.98	2.34	2.41	1.45	4.77
Disallowed (%)	0.07	0.00	0.07	0.07	0.00
